# Beneficial modulation of the gut microbiome by leachates of *Penicillium purpurogenum* in the presence of clays: A model for the preparation and efficacy of historical Lemnian Earth

**DOI:** 10.1371/journal.pone.0313090

**Published:** 2024-12-17

**Authors:** Simon Milling, Umer Zeeshan Ijaz, Danae Venieri, George E. Christidis, Nicholas J. W. Rattray, Iosifina Gounaki, Anna Andrusaite, Aravind Hareendran, Charles W. Knapp, Alexander X. Jones, Effie Photos-Jones

**Affiliations:** 1 Centre for Immunobiology, School of Infection and Immunity, University of Glasgow, Glasgow, United Kingdom; 2 Water and Environment Group, James Watt School of Engineering, University of Glasgow, Glasgow, United Kingdom; 3 School of Chemical and Environmental Engineering, Technical University of Crete, Chania, Greece; 4 School of Mineral Resources Engineering, Technical University of Crete, Chania, Greece; 5 Strathclyde Institute of Pharmacy and Biomedical Sciences, University of Strathclyde, Glasgow, United Kingdom; 6 Civil and Environmental Engineering, University of Strathclyde, Glasgow, United Kingdom; 7 Independent researcher, Antwerp, Belgium; 8 School of Humanities, University of Glasgow, Glasgow, United Kingdom; 9 Analytical Services for Art and Archaeology (Ltd), Glasgow, United Kingdom; South China Agricultural University, CHINA

## Abstract

The experiments presented here are based on the reconfiguration of an ancient medicine, Lemnian Earth (LE) (*terra sigillata*, *stamped earth*, *sphragis*), an acclaimed therapeutic clay with a 2500-year history of use. Based on our hypothesis that LE was not a natural material but an artificially modified one involving a clay-fungus interaction, we present results from experiments involving the co-culture of a common fungus, *Penicillium purpurogenum* (*Pp*), with two separate clay slurries, smectite and kaolin, which are the principal constituents of LE. Our results show: (a) the leachate of the *Pp*+smectite co-culture is antibacterial *in vitro*, inhibiting the growth of both Gram-positive and Gram-negative bacteria; (b) *in vivo*, supplementation of regular mouse diet with leachates of *Pp*+smectite increases intestinal microbial diversity; (c) *Pp+*kaolin does not produce similar results; (d) untargeted metabolomics and analysis of bacterial functional pathways indicates that the *Pp*+smectite-induced microbiome amplifies production of short-chain fatty acids (SCFAs) and amino acid biosynthesis, known to modulate intestinal and systemic inflammation. Our results suggest that the combination of increased microbial diversity and SCFA production indicates beneficial effects on the host microbiome, thus lending support to the argument that the therapeutic properties of LE may have been based on the potential for modulating the gut microbiome. Our experiments involving reconfigured LE open the door to future research into small molecule-based sources for promoting gut health.

## 1. Introduction

Lemnian Earth (LE) (*terra sigillata*, *stamped earth*, *sphragis*) was a highly prized therapeutic stamp-bearing clay, in the form of a pellet (S1 Fig), from Lemnos Island, North Aegean, Greece, with a documented record of near-continuous use, spanning 2500 years [1–8]. Its extraction, i.e. the digging out of the ‘earth’ at a particular locality on the northern shore of the island and subsequent ‘washing’ and preparation, at a nearby temple (in antiquity) or under the ‘blessing’ of the Church (in later times) was steeped in well-documented rituals (S1 File).

LE was taken orally, as a powder, on its own, drunk with wine, or mixed with botanicals; its distribution, both in antiquity and in the post-medieval and later periods, was tightly controlled. A close look at the medical literature (from 5^th^ c BCE to the late 19^th^ c CE) reveals that it was prescribed for several ailments over time, to include “bringing up blood” and ‘hemoptysis’ (“spitting of blood”); as an ‘antidote’ to poison ingested or venoms injected; as a preventative ‘against the plague’; and as a treatment for bowel issues (S1 Table). Nevertheless and despite its long-held and highly prized reputation, it has not been possible to explain what made LE, a seemingly ordinary clay, therapeutic.

In the past two decades, we have re-evaluated the existing evidence (documentary, geological, hydrological) associated with LE [9–14]. We have analysed historical samples of LE (16^th^-18^th^ c CE) in the collection of Basel University’s Museum of Pharmacy and compared their composition, bioactivity and microbial load with local clays [12, 13]. Historical samples and local clays were largely similar in composition and included kaolinite and smectite (montmorillonite). However, and regarding their bioactivity, whilst the historical samples were shown to be antibacterial against both Gram-positive and Gram-negative pathogens, the local clay was found to be non-bioactive. Regarding their microbial load (both bacterial and fungal), the historical samples showed evidence for clades of the Eurotiale phylogenetic order, i.e. *Penicillium and Talaromyces* phyla [13]. The natural clay showed none. The presence of these two *phyla*, as well as the scrutiny of select ancient texts and travellers’ accounts [9, 10, 14], led us to speculate that LE may have not been a natural clay but an artificially-modified one. For a summary explanation of the basis of this hypothesis, see S1 File.

The presence of a biome associated with a natural clay is not unknown; a Jordanian red clay [15] and the Kisameet Glacial clay, from Canada [16] were both found to contain bioactive ingredients and have been used, in this case, for healing skin diseases. In particular, the bioactivity of the Jordanian red clay was attributed to metabolites of actinomycetes bacteria (actinomycin C2 and actinomycin C3). What sets LE apart from these two clays is the presence of a fungal load which is absent in the natural clay from the same locality and which, we hypothesised, may have been introduced at first, accidentally, and subsequently, deliberately, in different ways at different times [9, 10, 14].

Ancient medical literature suggests that LE was always ingested. As such it could have an effect on the gut microbiome of the host, which in turn, is known to regulate both local gut health and systemic immune homeostasis [17]. The experiments presented here are based on the hypothesis that LE’s therapeutic properties may have pivoted around a fungus-clay interaction. The clays chosen had similar mineralogical compositions to those found in the historical samples, namely a smectite-rich clay, and a kaolinite-rich clay. The fungus chosen was *Penicillium purpurogenum*, a member of the *genera* identified in the historical samples [12, 13]. The purpose of the experiment was to establish the nature of the fungus+clay co-cultures, to assess the antibacterial activity of their leachates *in vitro*, and to monitor their effect on the mouse gut microbiome.

**Fig 1:**
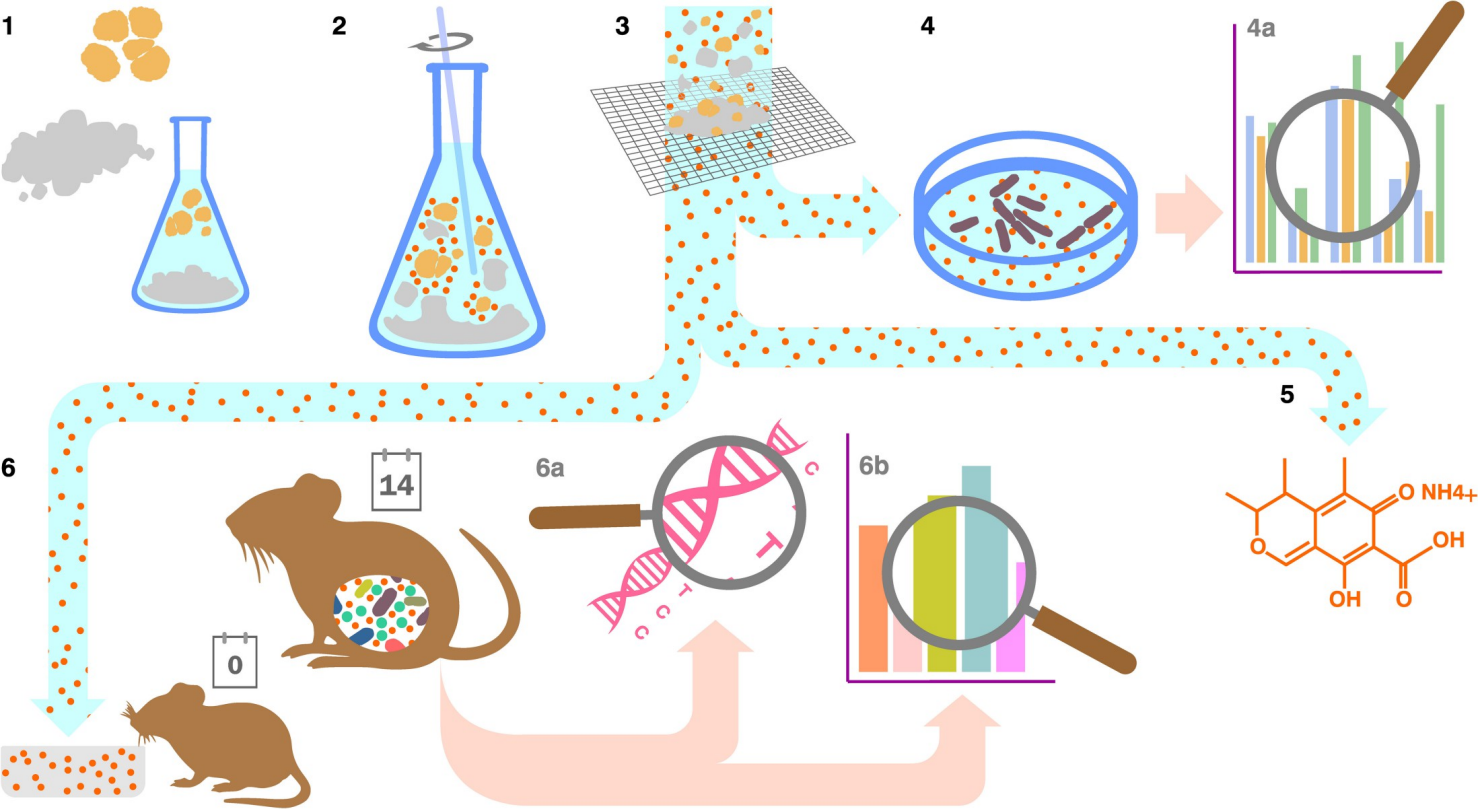
Illustration of the workflow of experiments and analyses of experimental results, as described in this paper. *Penicillium purpurogenum was* co-cultured with smectite or kaolin clays (2); co-cultures were filtered (3) and their leachates were tested for antibacterial activity *in vitro* (4) to establish reduction in Gram-positive and Gram-negative bacterial numbers (4a); this step was followed by targeted analysis of fungal metabolites present in each sample (5). The leachates of both co-cultures were then fed to mice, as a supplement to normal chow (6) during *in vivo experiments*; mouse stool was removed at day 0 and day 14. The effect of the leachates on the mouse microbiome was investigated via bacterial DNA sequencing (6a) and metabolomic analysis (6b).

With a specific combination of fungus and two different clays as the starting point, our experimental workflow is outlined as follows (Fig 1): co-cultures of the fungus and clays were prepared (step 2); they were subsequently filtered (step 3) and had the bioactivity of their leachates tested *in vitro*, against Gram-positive and Gram-negative bacteria (steps 4). The components of the leachates were identified by metabolomic analysis (step 5). The leachates were subsequently fed to mice to determine their effect on intestinal microbiota (step 6). The stool samples, at the start (day 0) and the finish (day 14) of the treatment, were analysed by DNA sequencing (step 6a) followed by metabolomic analysis (step 6b).

Our results demonstrate that the leachate of *Pp*+smectite increased microbial diversity in the gut, whereas the leachate of *Pp*+kaolin did not. In addition, only the leachate of the *Pp*+smectite culture preferentially modified the mouse intestinal microbiome via the increased production of short-chain fatty acids (SCFA) and aromatic amino acids (AAA). The paragraphs below serve as an introduction to the discussion section by helping to set the scene regarding the nature of the gut microbiome and its association with SCFAs.

A dysregulated microbiome (dysbiosis) is characterized by loss of bacterial diversity, altered metabolite production and colonization by toxic bacterial species [18]. It can be caused by several factors, including diet, environmental toxins, antibiotics, and stress. Dysbiosis is believed to be a contributing factor in the development of chronic diseases, including IBD (Crohn’s disease and ulcerative colitis) [19, 20].

Short chain fatty acids (SCFAs) including acetate (C2), propionate, (C3) and butyrate (C4) are end-products of the bacterial metabolism of dietary fibers [21]. They interact with intestinal epithelial and immune cell receptors as well as being absorbed and distributed systemically. Their net effect is to ameliorate local and systemic inflammatory pathways. SCFA deficits are biomarkers of gastrointestinal and inflammatory disorders [22]; they can benefit cardiometabolic health [23] and can serve as a potential treatment for lung infections [24]; as well as playing a critical role in brain-gut axis [25, 26].

At present, the major means for inducing increased SCFA production are via dietary supplements: probiotics (ingesting live cultures of microorganisms, including faecal transplants) and prebiotics (ingesting fiber-rich foods). Probiotic supplements help maintain a healthy gut microbiome by increasing the populations of bacteria (e.g. Lactobacilli) which assist digestion, outcompete opportunistic pathogens and synthesise metabolites (SCFAs and vitamins) for immune system regulation [27]. As a therapeutic mechanism, drawbacks include difficulty in bacteria surviving transit through the gastrointestinal tract, and transient effects due to a lack of stable colonisation [28]. Prebiotic dietary fibers can alter gut microbiome composition and are the substrate for SCFA production. However, predicting and controlling prebiotic outcomes is difficult as they influence multiple pathways and functions [28].

The work presented here is built on the hypothesis that LE’s therapeutic properties may have been based on a clay-fungus interaction. Our experimental results suggest that the addition of a clay mineral to fungal cultures produces a leachate rich in bioactive metabolites with beneficial properties. Furthermore, our experiments demonstrate how non-dietary-based supplementation may be used for microbiome modulation, with implications for intestinal health and immunological homeostasis.

## 2. Materials and methods

### a. Selection of the clays

Two samples supplied by the Clay Minerals Society, USA (CMS), namely SWy-2 smectite and KGa-2 kaolin, were added in the *Talaromyces* cultures. The specific clays were selected because a) they have been thoroughly characterized being part of the Source Clay Project of CMS and b) because smectite and kaolinite, the main constituents of the clays are also major components of the LE [12]. Smectite, the main constituent of SWy-2, is a 2:1 clay mineral consisting of two tetrahedral Si-sheets and one octahedral Al-sheet in between. Substitutions of Si by Al in the tetrahedral sheet and Al by Mg in the octahedral sheet create a negative layer charge, which is balanced by cations adsorbed in the space between the layers, known as interlayer space. Kaolinite, on the other hand, is a 1:1 clay mineral consisting of one tetrahedral Si-sheets and one octahedral Al-sheet. Substitutions in the lattice are negligible. Therefore, kaolinite does not bear layer charge.

### b. Preparation of leachates from clay+fungus co-cultures

The reference strain DSM 62866 of *Talaromyces purpurogenum* (formerly referred to as *Penicillium purpurogenum*–*Pp*, from the Leibniz Institute DSMZ· German Collection of Microorganisms and Cell Cultures) was used for microbiological analysis and the extraction of metabolites. *Talaromyces purpurogenum* was cultured in potato dextrose broth (Neogen), both with and without either SWy-2 smectite or KGa-2 kaolinite clay at 50 mg/mL. The cultures were incubated for 14 days at 25°C with agitation. The no-clay control had a pH of 5.1, whereas the SWy-2 and KGa-2 had pHs of 6.1 and 4.5, respectively.

Following the 14-day incubation, each sample (300 mL) was centrifuged at 10,000g for 20 min at 4°C. The supernatant was diluted in 100 mL of deionised water, boiled at 98°C for 15 min and then filtered through a 0.45 μm cellulose filter (Millipore, USA) to provide the *Pp* negative control (*Pp* control), *Pp*+smectite and *Pp*+kaolin filtrates. Notably, the thermal lysis and filtration steps were performed purely to rule out physical clay-bacteria interactions and the probiotic introduction of living fungi as mechanisms of bioactivity in vitro and in vivo.

### c. Measurement of in-vitro antibacterial activities of *Pp*+clay leachates

To determine their ability to inhibit the growth of bacteria, *Pp* control, *Pp*+smectite and *Pp*+kaolin filtrates were tested for their antibacterial properties against two reference bacterial indicators (Gram-negative) *Escherichia coli* DSM498 and (Gram-positive) *Staphylococcus aureus* NCTC 12493 (National Collection of Type Cultures, UK). The broth microdilution method was then used to estimate the Minimum Inhibitory Concentration that inactivated 60% of the bacterial population (MIC_60_). MIC values were estimated using 96-well sterile microtiter trays, which contained:

- dilutions of each liquid sample,
- LB broth (Neogen) and
- the bacterial population adjusted to 10^5^ CFU/mL.

The trays were incubated at 37°C for 18–24 h, followed by optical density measurement at 630 nm using a microplate reader (Labtech LT-4000 Plate Reader) and Manta LML software.

### d. Targeted fungal metabolomics

A targeted ultra-high performance liquid chromatography mass spectrometry (UHPLC-MS) approach was developed to qualitatively measure a list of 30 known bioactive secondary fungal metabolites (S2 Table). Chromatography was performed using a Thermo Vanquish UHPLC system (Thermo Fisher Scientific, Hemel Hempstead, UK) on a ZIC-pHILIC column (4.6 × 150 mm, 5μm particle size, Merck Sequant, Watford, UK). The column was maintained at 45°C and a flow rate of 300 μL/min. Mobile phases used were: (A) 20 mM ammonium carbonate and (B) acetonitrile. The gradient started with 20% (A) and increased to 95% (A) over 8 min then the composition was returned to its initial conditions in 2 min and the column was left to re-equilibrate at 400 μL/min for 4 min and then return to 300 μL/min in 1 min (15 min total time). Samples were kept at 4°C in the system autosampler, with 10 μL of extract being injected on to the column. A Thermo Exploris 240 mass spectrometer (Thermo Fisher Scientific, Bremen, Germany) was used in simultaneous ESI+ and ESI- modes for full LC-MS profiling and to generate data-dependent MS/MS (ddMS/MS) accurate mass spectra for identification of pigment masses provided within a targeted mass inclusion list. The operational parameters were as follows: spray voltage 3.9 kV (ESI+), 2.8 (ESI-), capillary voltage 20 V (ESI+), -15 V (ESI-), sheath, auxiliary and sweep gas flow rate were: 40, 5 and 1 (arbitrary unit), respectively, for both modes. Capillary and heater temperature were maintained at 275 and 150°C, respectively. Data were acquired in full scan mode with resolution 60,000 from m/z 70–1050. MS/MS scans on the 30 selected targets were performed at a resolution of 30,000 and a stepped normalized collision energy (NCE) of 10, 20 and 40.

### e. In-vivo feeding experiments

The *Pp*+smectite and *Pp*+kaolin leachates were administered to mice by gavage by a blinded researcher, to assess the effect of the filtrates on the intestinal microbiota of the mice. C57BL/6 mice were purchased from Envigo at 5–6 weeks of age and used for procedures at 7–8 weeks of age. Mice were housed under specific pathogen-free conditions at the University of Glasgow. All procedures were carried out under personal and project licenses issued by the UK Home Office.

Mice were administered 100 ml of PBS, *Pp*+smectite and *Pp*+kaolin leachates by oral gavage six times, every 2–3 days, over the course of two weeks. All animals received standard chow and sterile water ad libitum. The animals’ health was monitored daily, and their weight was measured every 2–3 days. Stool samples from individual mice were collected weekly, starting immediately before the first gavage, and were stored at -20°C before 16S and metabolomic analyses of the intestinal microbiota. At the end of the experiment, mice were humanely killed by exposure to a rising concentration of CO_2_. Death was confirmed by cervical dislocation.

### f. Mouse metabolomics

UHPLC-MS based untargeted metabolomics was performed on murine sample extracts where separation was performed on a binary Thermo Vanquish ultra-high performance liquid chromatography system. 5 μL of reconstituted extract was injected on to a Thermo Accucore HILIC column (100 mm × 2.1 mm, particle size 2.6 μm). The temperature of the column oven was maintained at 35°C, while the autosampler temperature was set at 5°C. For chromatographic separation, a consistent flow rate of 500 μL/min was used where the mobile phase in positive heated electrospray ionisation mode (HESI+) was composed of buffer A (10 mM ammonium formate in 95% acetonitrile, 5% Water with 0.1% formic acid) and buffer B (10 mM ammonium formate in 50% acetonitrile, 50% Water in 0.1% formic acid. Likewise, in negative ionization mode (HESI-), buffer A (10 mM ammonium acetate in 95% acetonitrile, 5% water with 0.1% acetic acid) and buffer B (10 mM ammonium acetate in 50% acetonitrile, 50% water with 0.1% acetic acid).

A high-resolution Exploris 240-Orbitrap mass spectrometer (Thermo Fisher Scientific) was used to perform full scan and fragmentation analyses. Global operating parameters were set as follows: spray voltages of 3900 V in HESI+ mode, and 2700 V in HESI-mode. The temperature of the transfer tube was set as 320°C with a vaporizer temperature of 300°C. Sheath, aux gas, and sheath gas flow rates were set at 40, 10, and 1 Arb, respectively. Data-dependent acquisitions (DDA) were performed using the following parameters: full scan range was 70–1050 m/z with a MS1 resolution of 60,000. Subsequent MS/MS scans were processed with a resolution of 15,000. High-purity nitrogen was used as nebulising and as the collision gas for higher energy collisional dissociation.

Data preprocessing ‐ Raw data was subsequently pre-processed to identify novel metabolic features within each sample class. RAW files obtained from Thermo Scientific Xcalibur 4.2 software and imported into Compound Discoverer 3.2 software where the “Untargeted Metabolomics with Statistics Detect Unknowns with ID Using Online Databases and mzLogic” feature was implemented. The workflow analysis performed retention time alignment, unknown compound detection, predicted elemental compositions for all compounds, and hides chemical background (using Blank samples). For the detection of compounds, mass, and retention time (RT) tolerance were set to 3 ppm and 0.3 min, respectively. The library search was conducted against the mzCloud, Human Metabolome Database (HMDB) and Chemical Entities of Biological Interest (ChEBI) database. A compound table was generated with a list of putative metabolites (known and unknown). Among them, we selected all the known compounds fully matching at least two of the annotation sources.

### g. Mouse microbiome statistics

Abundance tables were generated by constructing Amplicon Sequencing Variants (ASVs) using the QIIME2 workflow [29] using the DADA2 denoising algorithm [30]. Additionally, the PICRUSt2 algorithm [31] was used as a QIIME2 plugin on the ASVs to predict the functional abundance of microbial communities (both KEGG enzymes and MetaCyc pathways were recovered) by using the weighted Nearest Sequenced Taxon Index (NSTI) threshold of 2.0 in the software to map the ASVs against the reference database comprising ~20,000 genomes (whose functions were known) in PICRUSt2.

ASVs were then classified using the recent SILVA SSU Ref NR database release v.138 [32] and then combined the taxonomic information with the abundance table to generate a BIOM file. The rooted phylogenetic tree, also generated using the QIIME2 framework, along with the above BIOM file as well as the functional tables from PICRUSt2 were then used in the downstream statistical analyses in R.

As per the QIIME2 tutorials, typical contaminants were removed as a pre-processing step, such as Mitochondria, Chloroplasts and any ASVs that were unassigned at all levels. Samples that were irrelevant to this study (or are <2,000 reads) were also filtered out, giving an abundance table of n = 101 samples x P = 3,626 ASVs.

The summary statistics of sample-wise read distributions are as follows: Minimum: 62,504; 1st Quartile: 89,120; Median: 91,324; Mean: 89,950; 3rd Quartile: 95,460; Maximum: 98,607.

The R’s vegan package [33] was used for alpha and beta diversity analyses.

For alpha diversity measures rarefied richness was used–the estimated number of species/features in a rarefied sample (to minimum library size).

Different beta diversity distance measures used:

- Bray-Curtis distance on the ASV abundance table to visualise the compositional changes;
- Unweighted UniFrac distance estimated using R’s Phyloseq package [34] to see changes between samples in terms of phylogeny;
- Weighted UniFrac distance which also incorporates abundances and
- Hierarchical Meta-Storms (HMS) [35], a recent functional beta diversity distance that takes the observed KEGG Orthologs (KOs) recovered from the dataset, and then calculates the functional beta diversity distance in a hierarchical fashion propagating the KOs abundances upward to the pathways in a multi-level pathway hierarchy to give a weighted dissimilarity measure. R’s *aov()* function was used to calculate the pair-wise analysis of variance (*ANOVA*) with *p*-values drawn on top of alpha diversity.

To find a minimal subset of genera/pathways that changed between different conditions, the CODA LASSO [36] was used, in the form *y*_*i*_ = *β*_0_ + *β*_1_ log(*x*_1*i*_) + ⋯ + *β*_*j*_ log(*x*_*ji*_) + *ϵ*_*i*_ (for *i*-th sample and *j*-th microbe/pathway, with *x*_*ji*_ being the abundance of genera/pathway recovered from PICRUSt2), and where the outcome *yi* is a binary outcome variable (uses logistic regression).

The model uses two constraints:

- all *β*-coefficients sum up to zero which makes the algorithm invariant by returning two disjoint sets of features in a log contrast fashion, one that is positively associated and one that is negatively associated, and
- the optimization function incorporates a LASSO shrinkage term, which makes some *β*-coefficients go to zero with the non-zero *β*-coefficients returns for features (microbes/pathways) that change between the conditions. The coda *glmnet()* function was used from R’s coda4microbiome package [36]. The top 100 most abundant genera/pathways were used in the CODA-LASSO model.

## 3. Results

### 3a. In vitro bioactivity of *Pp*+clay leachates

As mentioned, *Pp* was grown in a potato dextrose broth (control) and then mixed with either smectite (SWY-2) or kaolin (KGa-2) at 50 mg/L. After two weeks, the co-cultured materials were centrifuged/filtered to separate the fungal and mineral components from the soluble leachates. These leachates were then used in the subsequent experiments to determine their ability to inhibit the growth of *Escherichia coli* and *Staphylococcus aureus*.

**Fig 2:**
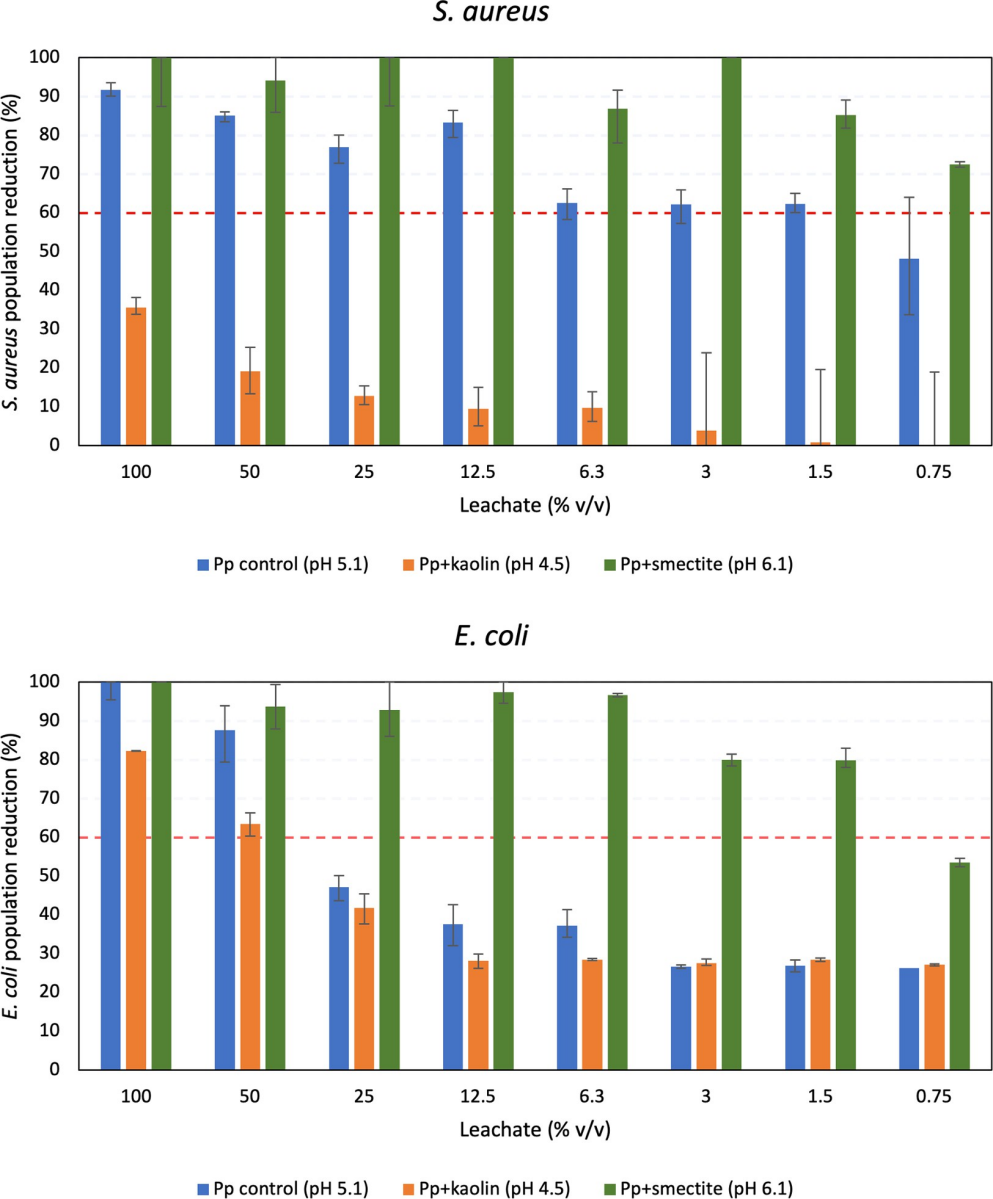
Reduction of *E*.*Coli* and *S*.*Aureus* populations upon incubation with the leachates of *Pp-*control, *Pp*+kaolin and *Pp*+smectite. The dashed red line indicates 60% reduction of each bacterial population.

The results of the testing of bioactivity of *Pp-*control (pH = 5.1), *Pp*+smectite (pH = 6.1) and *Pp*+kaolin (pH = 4.5) are shown in Fig 2. We selected to identify the minimum inhibitory concentration of the samples, which prevents visible growth of the test strains. This occurred only when 60% reduction of the initial population was recorded. The control leachate shows >60% reduction of *S*. *aureus* from 64x dilution (1.5% v/v leachate), and >60% inhibition of *E*. *coli* from 2x dilution. The leachate from the *Pp*+kaolin cultures is less effective: it inhibits >60% *E*. *coli* growth from 2x dilution and is ineffective against *S*. *aureus*. The *Pp*+smectite leachate shows the greatest antibacterial activity, inhibiting >60% growth of *E*. *coli* at 64x dilution and *S*. *aureus* at 128x dilution.

### 3b. Targeted LC-MS to identify potentially bioactive metabolites in *Pp*+smectite leachate

Fungi of the *Penicillium/Talaromyces* genera produce a variety of pigmented and non-pigmented secondary metabolites [37]. These have a range of bioactivities, including antibacterial, antifungal, and anti-inflammatory properties [38, 39]. We selected 30 of these pigmented secondary metabolites and biosynthetically related compounds (S2 Table). Examples of these metabolites, and their reported biological activities, are also provided (S3 Table) [40]. We performed targeted LC-MS/MS analysis of the *Pp*+clay leachates to assess their abundance (Table 1). Cyclopiazonic Acid, Monascorubrin, Purpurin, Rugulosin, ZG-1494a, Aversin showed zero counts for all three leachates.

**Table 1:** UHPLC-MS ion count values for targeted fungal pigment molecules.

	*MS Ion Counts*		
*MOLECULE*	*Pp* control	*Pp*+smectite	*Pp*+kaolin
*Ankaflavin*	1.55×10^8^	5.65×10^8^	4.54×10^5^
*PP-V*	0	2.77×10^6^	2.60×10^5^
*Purpuride*	1.56×10^5^	1.87×10^6^	1.35×10^7^
*PP-R*	2.13×10^5^	1.85×10^6^	1.32×10^6^
*Patulin*	0	1.65×10^6^	3.49×10^5^
*Purpogenic Acid*	3.30×10^5^	1.50×10^6^	0
*Mitorubrinol*	0	1.01×10^6^	1.81×10^5^
*Penicillic Acid*	2.71×10^6^	9.95×10^5^	2.29×10^6^
*Mitorubrin*	0	8.73×10^5^	0
*Roquefortine C*	6.09×10^4^	7.49×10^5^	0
*Rubropunctatin*	3.05×10^4^	5.48×10^5^	6.96×10^5^
*N-glutarylrubropunctamine*	0	4.56×10^5^	1.16×10^6^
*Monascin*	7.33×10^4^	3.11×10^5^	0
*Ochratoxin*	0	8.34×10^4^	0
*Monascorubramine*	9.16×10^5^	0	0
*Glauconic Acid*	7.76×10^5^	0	0
*Citrinin*	2.34×10^5^	0	5.67×10^5^
*N-glutarylmonascorubramine*	1.79E×10^5^	0	0
*Rubropunctamine*	2.94×10^4^	0	4.58×10^6^

Ten metabolites were identified that are most abundant in *Pp*+smectite compared to *Pp*+kaolin and *Pp*-control. It is suggested that these may be responsible for the observed *in vitro* antibacterial activity. Table 1 displays the observed ion counts for the targeted LC-MS/MS ranked by abundance in *Pp*+smectite. The ten compounds are highlighted, and heat maps show relative abundance to control and *Pp*+kaolin. All compounds were identified with Δ-values < 5 ppm. In addition, five of the compounds from the targeted list, i.e. PP-R, PP-V, purpuride, patulin, and citrinin could be identified *via* their MS/MS fragmentation patterns (S2 Fig).

One or more of these compounds enriched in *Pp*+smectite may be responsible for the observed *in vitro* antibacterial activity we observe. PP-R, PP-V, patulin, mitorubrinol and roquefortine C are known to possess antibacterial activity [41–43].Furthermore, ankaflavin, monascin and patulin are reported to show *in vivo* anti-inflammatory effects [44–47]. Due to its antibacterial activity, we hypothesised that the *Pp*+smectite leachate may have potentially beneficial effects mediated, at least in part, through modulation of the intestinal microbiota.

### 3c. In vivo microbiome analyses of *Pp*+clay leachates

**Fig 3:**
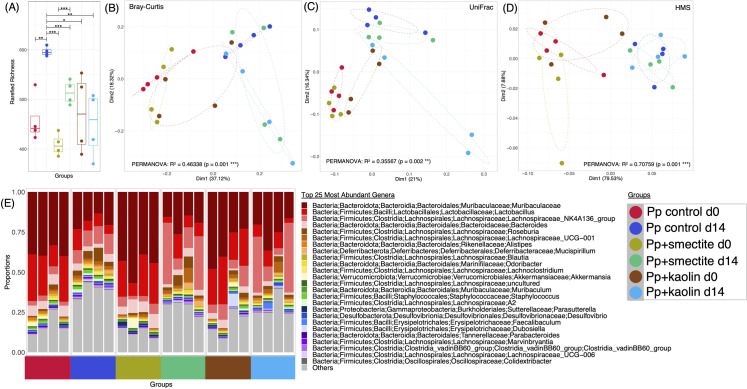
Diversity estimates for faecal microbiota from leachate-fed mice. Rarefied richness of bacterial ASVs shown in **(A)** with lines connecting different categories where values were significantly different (according to *ANOVA*; * p<0.05 ** p<0.01 *** p<0.001)., **(B-D)** show principal coordinate analysis (PcoA) plots with each axis showing the percentage variability explained by that axis, and where ellipses represent 95% CI of standard error for a given time point. Three distance matrices are used, Bray-Curtis **(B)** to reflect changes in composition, UniFrac (**C**) (to reflect changes in phylogeny), and HMS **(D)** (Hierarchical Meta-storms to reflect changes in function). (**E**) shows the top 25 most abundant genera recovered for each sample types.

To assess the potential for the *Pp*+clay leachates to modulate the intestinal microbiota, we opted to deliver them to mice, who also received chow and sterile water *ad libitum*. A control group received PBS, the other two groups received either *Pp*+smectite or *Pp*+kaolin leachate. Animals were checked daily, weighed weekly and displayed no signs of ill-health or weight loss. Faecal samples from each group of animals before and after two weeks of treatment were used for 16S sequencing, the results of which are summarised in Fig 3.

**Fig 4:**
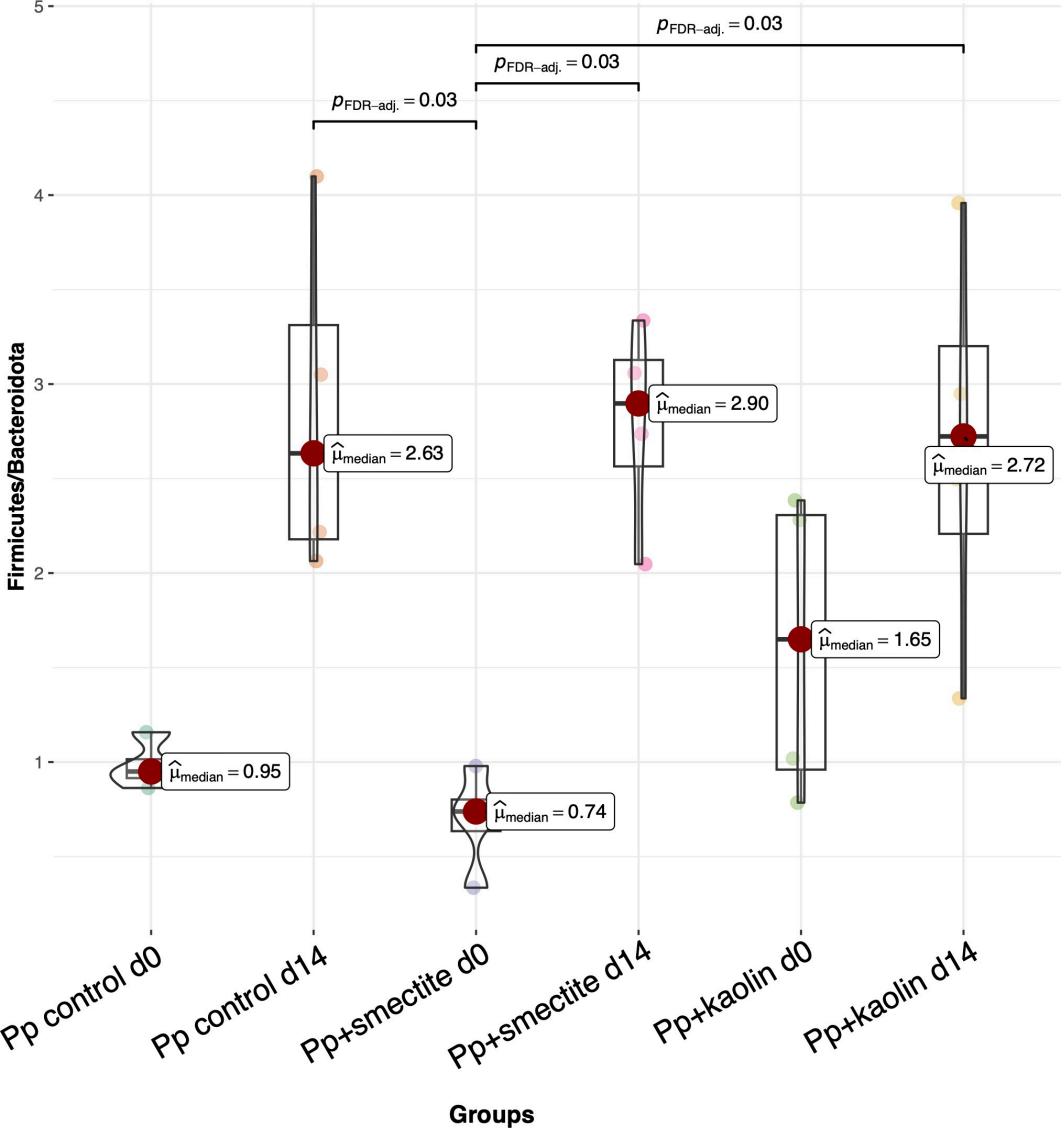
Firmicutes to Bacteroidetes ratio plotted using R’s ggstatsplot package [49] with ASVs collated at Firmicutes/Bacteroidetes level using the taxonomy identified through SILVA SSU Ref NR database release v.138.

For each sample, i.e. PBS, *Pp*+smectite, *Pp*+kaolin we applied CODA LASSO regression [36] to identify the significant genera which were altered in abundance before and after two weeks of feeding (Fig 3A–3E). Assessment of bacterial richness (Fig 3A) reveals that feeding with both PBS and *Pp*+smectite generated significant increases. Principle component analyses were also performed, using Bray-Curtis (Fig 3B), to reflect changes in composition, UniFrac (Fig 3C**)**, [34] to reflect changes in phylogeny, and Hierarchical Meta-storms (HMS) (Fig 3D), [35] to reflect changes in function. These again revealed significant changes after PBS or *Pp*+smectite feeding, but less consistent effects after feeding *Pp*+kaolin. Fig 3E shows the most abundant genera recovered from each group of animals. Both *Pp*+smectite and *Pp*+kaolin induce significant increases in firmicute *Lachnospiraceae* bacteria, while all samples reduce proportion of *Lactobacilli* and *Muribaculaceae* over the 14-day feeding bacteria. The overall changes in Firmicutes to Bacteroidetes ratio (F/B ratio), a biomarker implicated in gut dysbiosis, are shown in Fig 4 [48]. It is *Pp*+smectite which induces the greatest change in F/B ratio.

To better understand the significant biological changes that may be caused by the active fungal metabolites in the *Pp*+smectite leachate, we identified the bacterial genera with the highest positive and negative beta-coefficients after feeding with *Pp*+smectite. These are shown in Table 2 (see also S2 File). *β*-coefficients represent the weights associated with the log abundances of microbial genera. The procedure returns two sets of *β*-coefficients, those that are positively associated with d14 (i.e. increase in d14 as compared to d0), and those that are negatively associated with d14 (i.e., decrease in d14 as compared to d0). The highest positive changes were for *Lachnospiraceae*, *Desulfibrio and Eubacterium*, while a decrease in abundance after *Pp*+smectite was observed for *Muribaculum* and *Rikenellaceae*. These observed changes result from the combined effects of the mixture of bioactive fungal metabolites in the *Pp*+smectite leachate, which appear to favour particular members of the intestinal bacterial community, especially the *Lachnospiraceae*.

**Table 2:** Significant β-coefficients returned for *Pp*+smectite from the CODA-LASSO procedure.

*Bacterial Genera*	*β coefficient*	*Up*(+)/*Down*(-) *regulated*
*Incertae sedis*	0.09	+
*Lachnospiraceae*, *Lachnoclostridium*	0.03	+
*Lachnospiraceae*, *GCA-90066575*	0.03	+
*Desulfibrio*	0.03	+
*Eubacterium*, *siraeum*	0.02	+
*Lachnospiraceae*, *ASF536*	0	+
*Lachnospiraceae*, *Acetitafactor*	0	+
*Muribaculum*	-0.04	-
*Rikenellaceae RC9Gutgroup*	-0.09	-

Given that *Lachnospiraceae* are among the most abundant taxa in the gut microbiome [50], and were found to be the most significantly upregulated populations after feeding mice with *Pp*+smectite, we hypothesised that they may have an important effect on the host animals. All members of the *Lachnospiraceae* are anaerobic, fermentative and chemo-organotrophic [50, 51]. *Lachnospiraceae* are also among the main producers of short-chain fatty acids (SCFAs) from metabolism of dietary fibers/starch [52]. SCFAs could, therefore, provide a mechanism by which the significant changes in the microbiota that occur after feeding *Pp*+smectite could be beneficial for the intestinal environment. We, therefore, performed untargeted metabolomics to determine whether SCFAs were present at increased levels in any of our samples.

### 3d. Untargeted metabolomic analysis of murine stool samples

Untargeted murine metabolomics of stool samples did not directly reveal the presence of acetate, propionate, butyrate, or C5 SCFAs. This may be due to their volatility and low molecular weight, and the fact that our sample preservation protocols were not optimised for the preservation of volatile molecules. However, SCFA conjugates with carnitine, an SCFA transporter, were detected [53].

**Fig 5:**
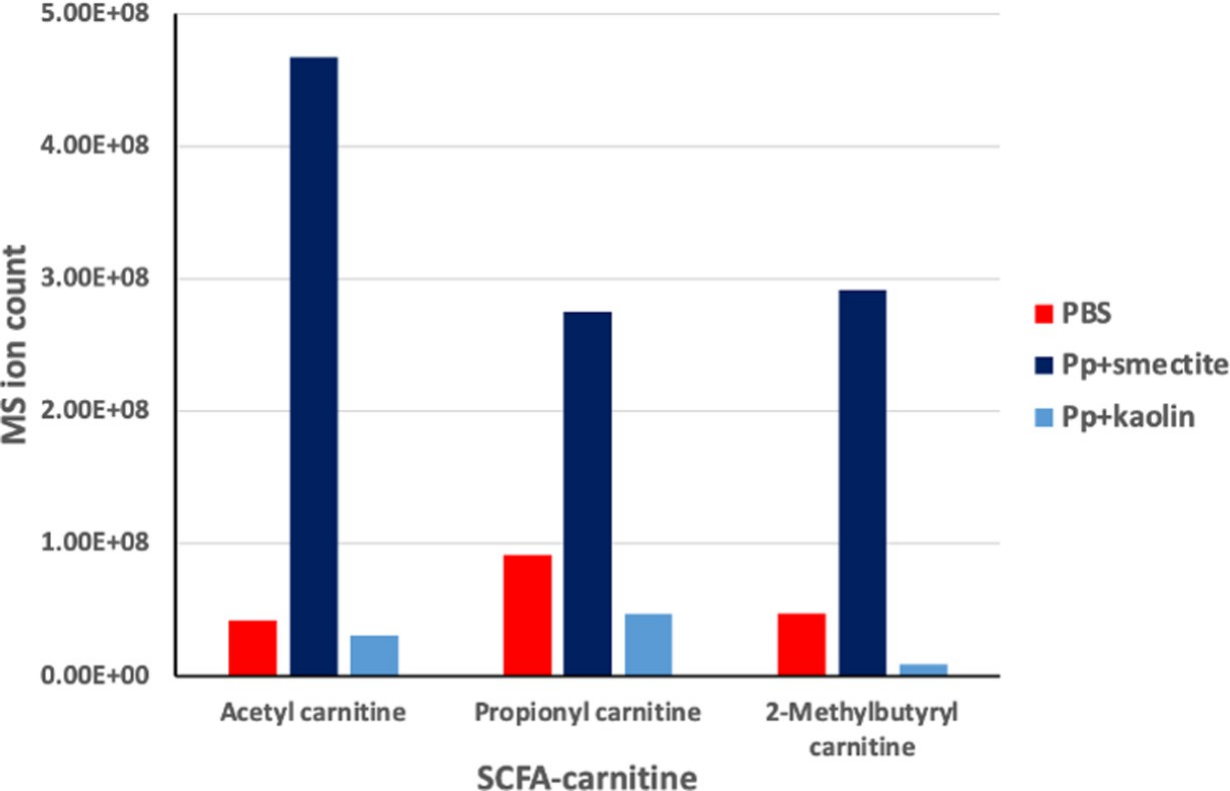
MS ion counts of SCFA conjugates with carnitine identified in murine stool samples when fed with *Pp*+smectite, *Pp*+kaolin and PBS.

These conjugates are less volatile, and so were preserved in our samples. Importantly, Acetyl-, propionyl-, and 2-methylbutyryl-, carnitine were all identified with much higher intensities in samples from mice fed with *Pp*+smectite relative to the control and *Pp*+kaolin samples (Fig 5). It appears, therefore, that the abundance of SCFAs in the intestines of mice fed with *Pp*+smectite were indeed higher than in the mice from the other groups.

The changes in microbial populations that occur after feeding mice with *Pp*+smectite may drive metabolic changes in the intestinal lumen beyond the changes in SCFAs. In order to assess such changes, we performed MetaCyc pathway analysis to impute bacterial functional metabolic pathways from our 16S datasets **(**Table 3). Following pathway analysis, upregulated and downregulated pathways unique to *Pp*+smectite feeding were identified using CODA LASSO regression [36]. Consistent with our identification of SCFA-carnitine adducts in our *Pp*+smectite samples, upregulation of the GLYCOCAT pathway for glycogen degradation showed a significant increase in Centralised Log Ratio and beta-coefficient (0.12) **(**Table 3); see also S3 File [52]. The unique upregulation of GLYCOCAT matches well with the increased SCFA production noted in *Pp*+smectite.

**Table 3:** Up-regulated and down-regulated pathways unique to *Pp*+smectite with positive β-coefficients increase in d14 as compared to d0.

*Up-regulated pathways unique to Pp*+smectite	
*Pathway*	*β coefficient*
*S-adenosyl-L-methionine biosynthesis*	0.19
*glycogen degradation I*	0.12
*methylerythritol phosphate pathway I*	0.06
*L-glutamate and L-glutamine biosynthesis*	0.05
*NAD salvage pathway I*	0.04
*cis-vaccenate biosynthesis*	0.05
*chorismate biosynthesis from 3-dehydroquinate*	0.02
*L-isoleucine biosynthesis III*	0.01
*L-ornithine biosynthesis*	0
*adenine and adenosine salvage III*	0
*L-arginine biosynthesis*	0
*superpathway of purine nucleotides de novo biosynthesis I*	0
*superpathway of purine nucleotides de novo biosynthesis II*	0
*gluconeogenesis I*	0
***Down-regulated pathways unique to Pp*+smectite**	
***Pathway***	***β coefficient***
*gondoate biosynthesis (anaerobic)*	-0.01
*superpathway of guanosine nucleotides de novo biosynthesis II*	-0.02
*superpathway of pyrimidine ribonucleosides salvage*	-0.02
*incomplete reductive TCA cycle*	-0.02
*superpathway of pyrimidine nucleobases salvage*	-0.04
*fatty acid elongation—saturated*	-0.19
*GDP-mannose biosynthesis*	-0.27

In addition to changes in SCFA-related pathways, other pathways were also identified as being significantly upregulated or downregulated uniquely after *Pp*+smectite treatment; these changes are summarised in Table 3. Among the significant changes, we observed that several anabolic amino acid pathways are upregulated after *Pp*+smectite, including glutamate and glutamine biosynthesis; tryptophan, tyrosine and phenylalanine via chorismite biosynthesis; as well as isoleucine and ornithine and arginine biosynthesis. Overall, we deduce that the effect of *Pp*+smectite on bacterial metabolism in the intestine may be to increase both the availability to the host of SCFAs, and of amino acids.

Increasing evidence indicates that AA metabolism in the small intestine plays important roles in the regulation of whole-body AA homeostasis [54]. AA metabolites in the gut also play key roles in immune system modulation and cytokine responses. Metabolites of aromatic AAs (tryptophan, tyrosine) are ligands of the aryl hydrocarbon receptor (AHR), which is a ligand-dependent transcription factor that controls immune responses in the intestine, and is critical for maintaining barrier functions [55].

The majority of downregulated functional pathways in *Pp*+smectite are associated with reduced *de novo* biosynthesis and salvage of nucleotides: superpathway of guanosine nucleotides de novo biosynthesis II; superpathway of pyrimidine ribonucleosides salvage; and superpathway of pyrimidine nucleobases salvage. Modulation of nucleotide biosynthetic pathways within the complex gut environment, and their interactions with the host immune system have not been explored to the same extent as AAs and SCFAs [56].

## 4. Discussion

In this work, we selected two inert reference clay samples, SWY-2 smectite and KGa-2 kaolin, to reconfigure the ancient LE production process. The co-cultures of the individual clays with *Penicillium Purpurogenum (Pp)* were examined by targeted fungal metabolomics. We demonstrate, for the first time, that *Pp*+smectite and *Pp*+kaolin differentially alter the quantity and distribution of bioactive secondary metabolites produced, both relative to each other and in comparison to cultures without clay.

In addition, the *Pp*+smectite leachate is significantly more antibacterial *in vitro* against *E*. *coli* and *S*. *aureus* than *Pp*+kaolin and the control broth. After screening the literature for secondary metabolites produced by *Pp* and similar genera possessing antibacterial, antifungal and/or anti-inflammatory activities, thirty metabolites were selected from biosynthetic classes, including azaphilones, anthraquinones and common mycotoxins. Compared to *Pp* control, *Pp*+smectite induced a significant enrichment in monascin and ankaflavin (anti-inflammatory, anti-cancer) [44, 45]; mitorubrin and mitorubrinol (antibacterial) [57]; *PP*-V and *PP*-R (antibacterial, antifungal) [41, 58]; roquefortine C (antibacterial) [59–61]; and the toxins patulin [62–64] and ochratoxin. Other metabolites were reduced, *e*.*g*. monascorubramine, penicillic acid and rubropunctamine. By contrast, *Pp*+kaolin induced enrichment in rubropunctatin and its derivatives rubropunctamine and N-Glutarylrubropunctamine [65].

The mechanism by which smectite and kaolinite induce such differential metabolite expressions in *Pp* is likely related to the different surface properties of the two clay minerals. Na-smectites, like the SWy-2, have a negative layer charge due to ionic substitutions in the lattice that is balanced by the interlayer cations. In addition, they swell upon hydration due to the introduction of water molecules that hydrate the interlayer cations in the interlayer space. The latter are exchangeable, thereby yielding significant cation exchange capacity (CEC) for inorganic cations and organic molecules [66]. In contrast, kaolinite does not have permanent layer charge and thus it bears very low CEC due to unsatisfied bonds at crystalline edges. The negative charge of SWy-2 crystallite surfaces may repel negative membrane charge of *Pp*. Simultaneously, smectite readily adsorbs molecules from the agar nutrient (potato dextrose) in the interlayer, thereby increasing the interlayer space, i.e. the distance between the smectite layers. Such a process will increase swelling and will facilitate delamination of Na-rich SWy-2 lamellae, thereby increasing the area of contact between smectite and *Pp*, and thus intensifying the clay-fungus interaction.

In other words, the smectite surfaces could act as stressors on the *Pp*, triggering increased production of secondary metabolites. Such an interaction between clay mineral and *Pp* was not observed in kaolinite because of the lack of permanent charge and lack of swelling of the crystals.

Having established that *Pp*+smectite leachate displayed antibacterial activity *in vitro*, we proceeded to test it *in vivo*. It was important to rule out physical clay-bacteria interactions and probiotic introduction of living fungi as mechanisms of bioactivity in the murine intestine. Therefore, we performed all *in vivo* experiments with the leachates of the co-cultures, after thermal carrying out lysis and filtration.

We analysed the faecal microbiota of mice after feeding leachates. *Pp+*smectite induced surprising and potentially health-promoting effects in intestinal bacteria, including increases in bacterial diversity and an increase in the ratio of Firmicutes/Bacteriodetes bacteria (F/B ratio). Although the F/B ratio is no longer considered a quantitative biomarker of dysbiosis in the clinic, a reduced F/B ratio is commonly correlated with IBD [48, 67]. There have been multiple reports of treatment of IBD with probiotics which increase the F/B ratio to restore a healthy state [67].

We also observed a significant increase in bacteria that produce SCFAs, particularly the Lachnospiraceae. Untargeted metabolomics of the faecal metabolites revealed the increased formation of SCFA-carnitine adducts, consistent with an increased abundance of volatile SCFAs *in vivo*. Functional pathway analysis of the post-feeding microbiome was consistent with SCFA production *via* upregulated glycogen degradation and revealed increased AA biosynthesis. Several reports of small-molecule-induced microbiome remodelling cite similar evidence: cyclic peptides were shown to induce SCFA and AA upregulation and beneficial effects in an atherosclerotic mouse model [68].

The results of our *in vivo* analyses point to several paths forward for interrogating mechanistic questions and identifying relevant disease models. Future work will focus on elucidating the active ingredient(s) within the leachate responsible for *in vitro & vivo* observations. Interactions between these active ingredients, the intestinal microbiota, and the mouse immune response will also be analysed in disease and infection models, to identify additional therapeutic effects of the leachates.

## 5. Conclusions

We have described a series of carefully characterised laboratory experiments to interrogate the biological effects of *Pp*+clay leachates. We have demonstrated that:

- Co-cultures of *Pp* with clays induce a differentiated profile of secondary metabolite expression. The mechanisms by which clays stress fungi into producing metabolites are presently unclear and will be subject for further investigation.
- The leachate of the *Pp*+smectite culture showed antibacterial activity against both Gram-positive and Gram-negative, whereas the *Pp*+kaolin leachate was not bioactive.
- When the different *Pp*+clay cultures were tested *in vivo*, only the leachate of the *Pp*+smectite culture modified the mouse intestinal microbiome and the production of short-chain fatty acids (SCFA) *and* amino acids (AAs). SCFAs modulate intestinal and systemic inflammation and convey health benefits to the host.

In summary, we have shown that one leachate from the fungus+clay co-cultures modified the intestinal microbiota, thus enhancing the production of SCFAs. This is one of an increasing number of indications that non-diet-related, small molecule supplementation may be successful in maintaining intestinal health and immunological homeostasis.

Regarding the LE, the precise recipe for its preparation remains, and is likely to remain, unclear and further, it may have been modified over time. Investigating LE, and what has been known about it, has served as a springboard to investigate the modulation of the microbiome in a targeted fashion. Our data provide a potential mechanism by which fungus+clay co-cultures may be a valuable tool for manipulating the microbiota to prevent the progression of inflammatory diseases, and perhaps also limit intestinal infections; it therefore suggests avenues for the further development of ancient LE’s potential in a 21^st^ century context.

## Supporting information

S1 Fig Historic Lemnian Earth (LE). (PDF)

S2 Fig MS/MS spectra of identified fungal secondary metabolites. (PDF)

S1 Table Selection of excerpts from the medical literature (300 BCE-19^th^ CE) referring to LE. (PDF)

S2 Table Fungal secondary metabolites selected for targeted metabolomics. (PDF)

S3 Table A summary of the antimicrobial and anti-inflammatory properties exhibited by fungal secondary metabolites encountered in our experiments. (PDF)

S1 File Ancient Lemnian Earth (LE) or *sphragis* and the basis of a hypothesis. (PDF)

S2 File Analysis of mouse microbiome bacterial taxa. (PDF)

S3 File Analysis of mouse microbiome functional pathways. (PDF)
