# Supplementary material for: Beneficial modulation of the gut microbiome by leachates of Penicillium purpurogenum in the presence of clays: A model for the preparation and efficacy of historical Lemnian Earth
Source: PLoS One. 2024 Dec 17;19(12):e0313090. doi: 10.1371/journal.pone.0313090 (PMC11651545; doi:10.1371/journal.pone.0313090)

Figure S1: Historical Lemnian Earth. Specimens of Lemnian Earths (terra sigillata, *sphragis*) in the collections of the University of Basel, Museum of Pharmacy, dated 16th-18th c [12]. (a) Lemnian *sphragis* (red). MA no. 01422; (b) Lemnian *sphragis* (grey). MA no. 01424; (c) Lemnian *sphragis* (white). MA no. 01432.

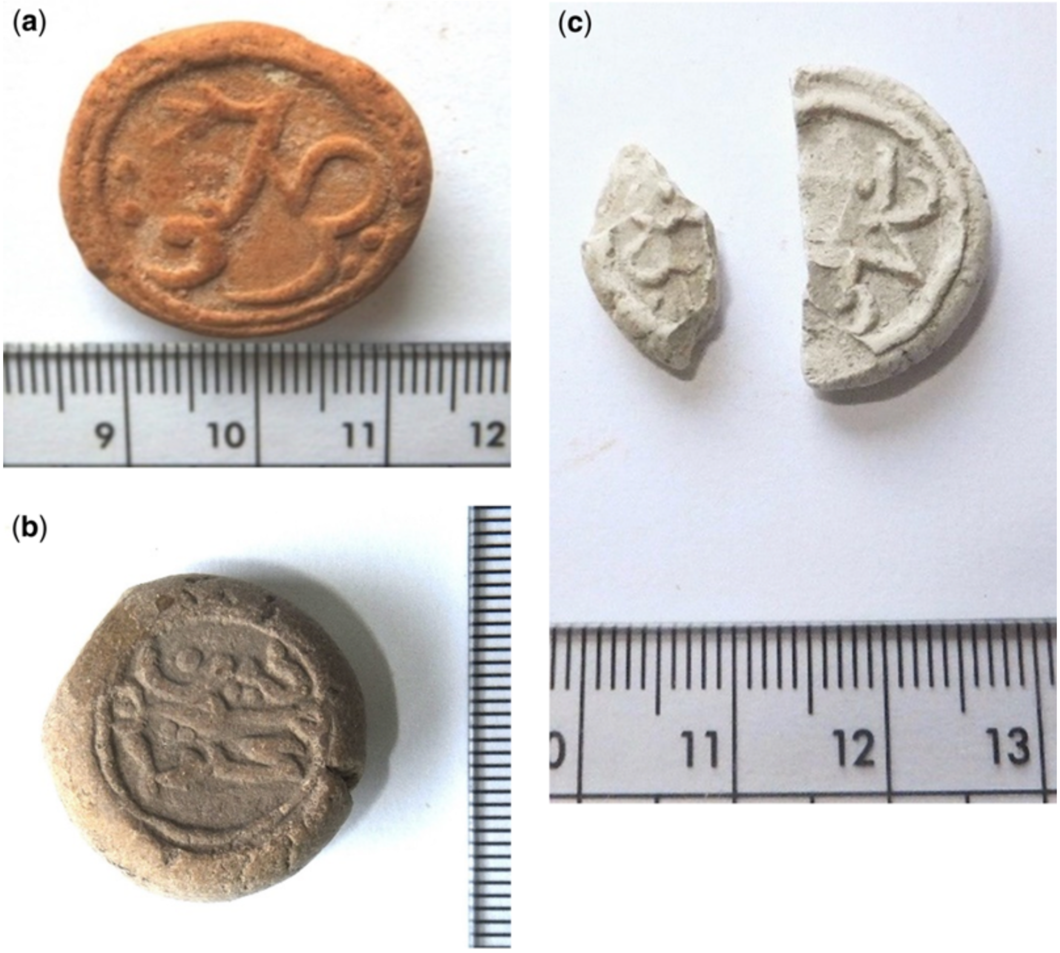

Supplement: S1 Fig — (PDF) [file pone.0313090.s001.pdf]
