# Supplementary material for: Beneficial modulation of the gut microbiome by leachates of Penicillium purpurogenum in the presence of clays: A model for the preparation and efficacy of historical Lemnian Earth
Source: PLoS One. 2024 Dec 17;19(12):e0313090. doi: 10.1371/journal.pone.0313090 (PMC11651545; doi:10.1371/journal.pone.0313090)
Supplement: S2 Fig — (PDF) [file pone.0313090.s002.pdf]

Figure S2: MS/MS Spectra of Identified Fungal Secondary Metabolites

Citrinin

citrinin

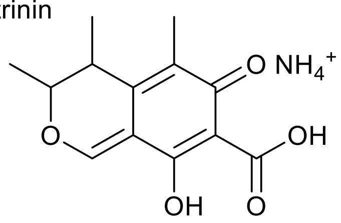

Chemical Formula: $C_{13}H_{18}NO_5^+$
Exact Mass: 268.1179

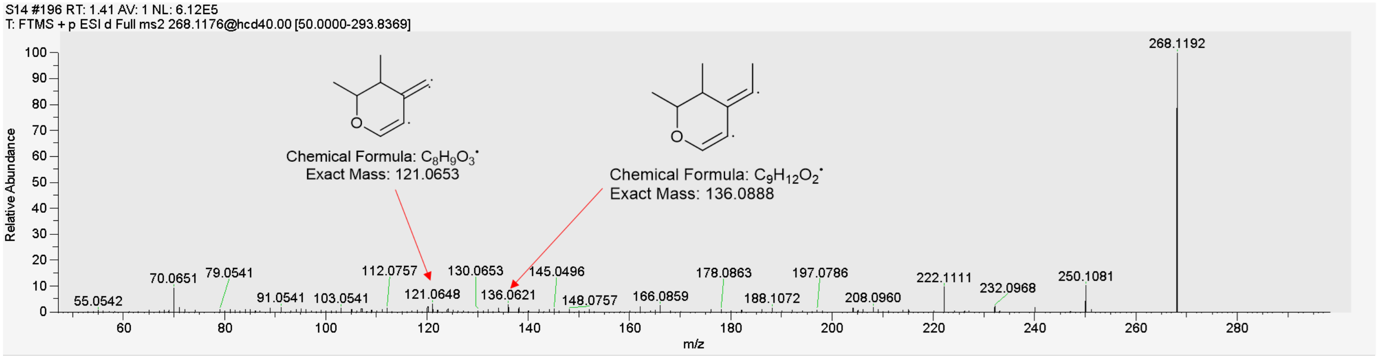

Purpuride

Chemical Formula: $C_{22}H_{34}NO_5^+$
Exact Mass: 392.2431

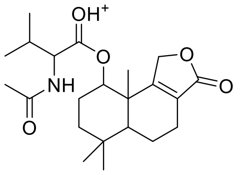

purpuride

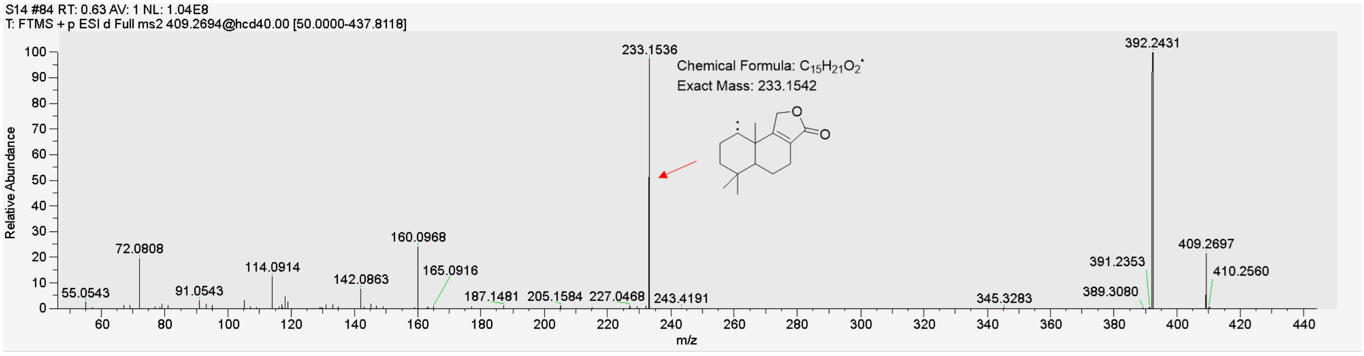

Patulin

Chemical Formula: $C_7H_5O_4^-$
Exact Mass: 153.0193

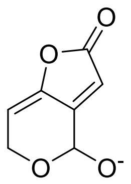

patulin

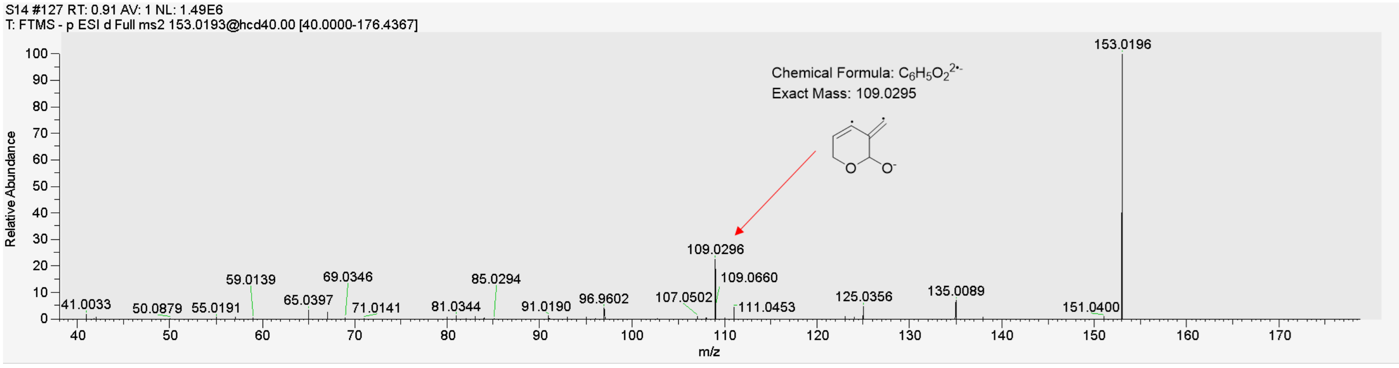

PP-R

Chemical Formula: $C_{25}H_{35}N_2O_5^+$
Exact Mass: 443.2540

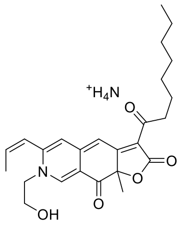

PP-R

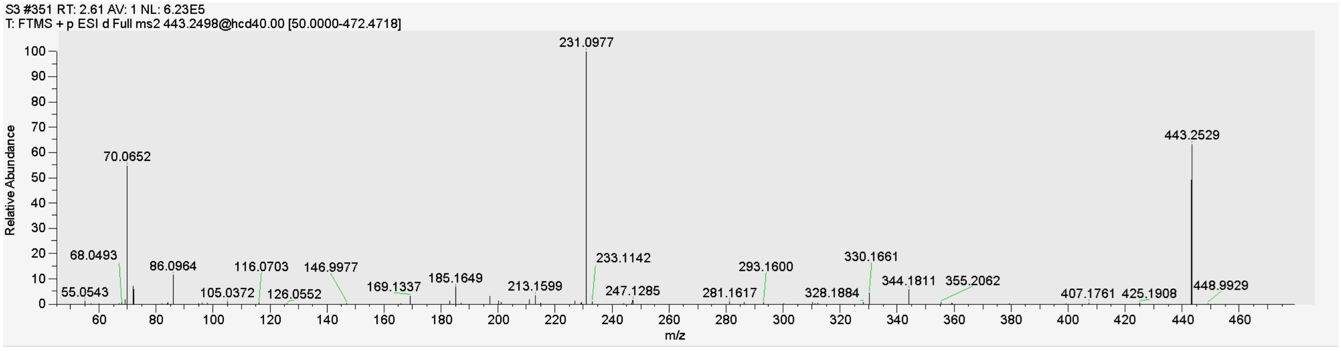

PP-V

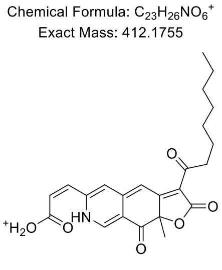

Chemical Formula: $C_{23}H_{26}NO_6^+$
Exact Mass: 412.1755
O
HN
O
O
O
^+H_2O
O
PP-V

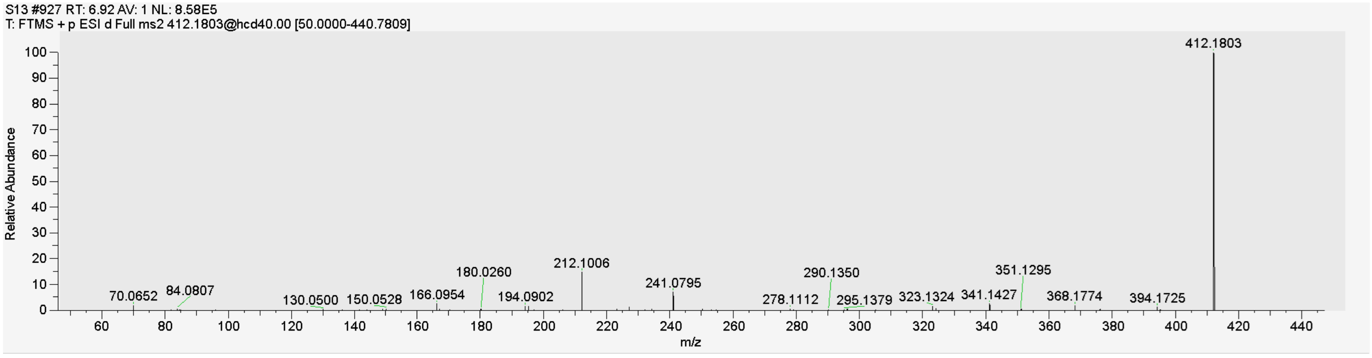

S13 #927 RT: 6.92 AV: 1 NL: 8.58E5
T: FTMS + p ESI d Full ms2 412.1803@hcd40.00 [50.0000-440.7809]
Relative Abundance
m/z
412.1803
212.1006
241.0795
180.0260
290.1350
351.1295
70.0652
84.0807
130.0500
150.0528
166.0954
194.0902
278.1112
295.1379
323.1324
341.1427
368.1774
394.1725
