# Supplementary material for: Beneficial modulation of the gut microbiome by leachates of Penicillium purpurogenum in the presence of clays: A model for the preparation and efficacy of historical Lemnian Earth
Source: PLoS One. 2024 Dec 17;19(12):e0313090. doi: 10.1371/journal.pone.0313090 (PMC11651545; doi:10.1371/journal.pone.0313090)
Supplement: S1 Table — (PDF) [file pone.0313090.s003.pdf]

Table S1: Selection of excerpts from the medical literature (300 BCE-19th CE) referring to LE.

The table below gives a selection of ailments for which LE was prescribed in antiquity and later periods, as extracted from a compilation of such references by Paximadas [8] with translations into English by EP-J. As can be seen, the use of LE could be more specific than the oft-repeated attribute i.e. 'antidote against poison'; it relates particularly to the bringing up of blood probably associated with the upper gastrointestinal tract (mouth, oesophagus, stomach and upper small intestine) and/or the lungs. The frequent reference to the epiglottis, uvula, tonsils is also of interest.

**Author**	**Date**	**Therapy for**	**In Greek**
Herophilos	c. 300BCE	the bringing up of blood	*προς αίματος αναγωγήν*
Apollonius of Memphis	c. 300BCE		*προς τα εν ωσίν υπερσαρκώματα*
Aretaeus of Cappadocia	2nd c BCE	the bringing up of blood	*προς αίματος αναγωγήν*
Asklepiadis	2nd c BCE	those with stomach problems	*στους στομαχικούς*
Crateroς the Roman		those who have spat blood, a very good tonic	*προς αιμοπτυικούς ευτονώτατον*
Apollonius Mys	1st c BCE	the bringing up of blood; those who suffer from colic' a preventive antidote;	*προς αίματος αναγωγήν. κωλικήν. αντίδοτον προφυλακτική*
Servilius Damocrates (or Democrates)	1st c BCE	those who suffer from chronic consumption, it is strongly effective	*σφόδρα καλή προς φθισικούς κεχρονισμένους*
Dioscorides	1st c CE	conditions that concern the lungs and the thorax; conditions that concern the intestines; conditions of the gall bladder; conditions that concern the liver and spleen.	*περί τον πνεύμονα και θώρακα παθών. των περί τα έντερα παθών. περί την κύστην χολήδοχον. ήπαρ τε και τον σπλήνα παθών*
Pliny the Elder	1st c CE	antidote against poison; vomiting blood or spitting of blood.	
		It is an uncommonly effective antidote for deadly poisons when drunk with wine, and, when taken ahead of time, it forces one to vomit the poisons. It is suitable both for the strokes of venomous animals and for their bites. It is mixed with antidotes.	
Galen	2nd c CE	for bringing up blood, for those who spit blood; for wounds that are malignant and festering, every time I used the LE, it was greatly effective; the amount used was dependent on the severity of the wound	
Oribasius	4th c CE	conditions of the bladder; for those who bleed during an operation; those who spit blood; those who bring up blood; those with dysentery; those who suffer in the bowels; those with malignant, chronic and hard to cicatrize wounds	
Alexander of Tralles	6th c CE	for sore throat; for dysentery΄for bringing up blood	
Theophanes Nonos	10th c CE	for conditions of the uvula; for spitting of blood	
Bartholomaeus Anglicus	13th c CE	for noose bleeds as a haemostatic; for swollen parts of the body (feet)	
Agricola Georgius	16th c	'according to the Turks, the only medicine against the plague'	
Pierre Bellon	16th c	a preventive against the plague and other epidemics	
Tommaso Porcacchi	16th c	excellent medicine for the curing of wounds that fester, maladies that do not recover, bites of poisonous animals and to help relieve the sick from poisons already ingested	
Olfert Dapper	17th c	a medicine prescribed for wounds inflicted, bites of poisonous snakes, gives back clotted blood its natural flow; strengthens the heart and induces sweating. Its main use is in malignant fevers, the plague, dysentery and the bites of poisonous snakes and insects	
Robert Walpole	18th c	a strong medicine against severe fevers	
