# Supplementary material for: Beneficial modulation of the gut microbiome by leachates of Penicillium purpurogenum in the presence of clays: A model for the preparation and efficacy of historical Lemnian Earth
Source: PLoS One. 2024 Dec 17;19(12):e0313090. doi: 10.1371/journal.pone.0313090 (PMC11651545; doi:10.1371/journal.pone.0313090)
Supplement: S2 Table — (PDF) [file pone.0313090.s004.pdf]

Table S2.1: Fungal secondary metabolites selected for targeted metabolomics

MOLECULE
Ankaflavin
PP-V
Purpuride
PP-R
Patulin
Purpogenic Acid
Mitorubrinol
Penicillic Acid
Mitorubrin
Roquefortine C
Rubropunctatin
N-glutarylrubropunctamine
Monascin
Ochratoxin
Monascorubramine
Glauconic Acid
Citrinin
N-glutarylmonascorubramine
Rubropunctamine
Cyclopiazonic Acid
Monascorubrin
Purpurin
Rugulosin
ZG-1494a
Aversin
Secalonic acid
Purpurogenone
Rubratoxin B
Limonene
