# Supplementary material for: Beneficial modulation of the gut microbiome by leachates of Penicillium purpurogenum in the presence of clays: A model for the preparation and efficacy of historical Lemnian Earth
Source: PLoS One. 2024 Dec 17;19(12):e0313090. doi: 10.1371/journal.pone.0313090 (PMC11651545; doi:10.1371/journal.pone.0313090)
Supplement: S3 Table — (PDF) [file pone.0313090.s005.pdf]

Table S2.2: A summary of the antimicrobial and anti-inflammatory properties exhibited by fungal secondary metabolites encountered in our experiments **[40].**

**Metabolite**	**Antimicrobial**				**Anti-inflammatory**
	**Antibacterial**	**Antifungal**	**Antiviral**	**Antiprotozoal**	
Citrinin	+	+	-	-	+
Ankaflavin	-	-	-	-	+
Monascin	-	-	-	-	+
Cyclopiazonic acid	+	-	+	-	-
Mitorubrinic acid	+	+	-	+	+
Mitorubrinol	+	-	-	+	-
Monascorubrin	+	+	-	-	-
OTA	-	-	-	-	+
Patulin	+	-	-	-	+
Penicillic acid	+	-	-	-	-
PP-V and PP-R	+	+	-	-	-
Roquefortine	+	-	-	-	-
Rubropunctamine	+	-	-	-	-
ZG-1494α	+	-	-	-	+
Limonene	+	-	-	-	+

(+ Activity present, - Activity absent)
