# Supplementary material for: Beneficial modulation of the gut microbiome by leachates of Penicillium purpurogenum in the presence of clays: A model for the preparation and efficacy of historical Lemnian Earth
Source: PLoS One. 2024 Dec 17;19(12):e0313090. doi: 10.1371/journal.pone.0313090 (PMC11651545; doi:10.1371/journal.pone.0313090)
Supplement: S1 File — (PDF) [file pone.0313090.s006.pdf]

File S1. ***Ancient Lemnian Earth (LE) or sphragis and the basis of a hypothesis.***

The most detailed account we have of the events on the day of LE's extraction come from no less a figure than Galen, the renowned doctor of the Greco-Roman world. He visited the island in the late second century CE and gave an insightful account of LE's extraction and processing in his book *On the Properties of Simple Drugs.* He described the ritual of the preparation of the *Lemnian Earth (sphragis)* as follows:

The priestess collects the clay to the accompaniment of some local ceremony no animals being sacrificed, but wheat and barley being given back to the land in exchange. She then takes it to the city, mixes it with water to make moist mud, shakes this violently and then allows it to stand. Thereafter she removes first the superficial water, and next the greasy part of the earth below this, leaving only the stony and sandy part at the bottom, which is useless. She now dries the greasy mud until it reaches the consistency of soft wax; of this she takes small portions and imprints upon them the seal of Artemis; then again, she dries these in the shade till they are absolutely free from moisture [3].

Of interest in his description are the following points: a. the addition of wheat and barley to the clay that has been extracted; b. the addition of water and vigorous mixing of clay+ wheat/barley; c. the sequential settling of 'clay' of various consistencies ('moist mud', 'greasy earth', 'stony/sandy part'). In our publications [9,10,14] we have speculated that if the clay extraction took place in May, as it is reported to have been the case (in the Christian period it took place in August, instead) the grain used would have been of the last season's and as such some of it may have been fungus infested. Indeed, the phyla *Aspergillus* and *Penicillium* which are known common fungal contaminants in stored grain were found to be present in some of the historical samples we analyzed [13]. In the post medieval period, there is no specific reference to 'blessing' by the Christian clergy with wheat and barley; although this practice cannot be excluded, other ways of enriching the clay in fungal growth (like keeping the pit under water over prolonged periods of time) have been reported. Notable amongst others is a 15th c eyewitness observation by a Dutchman visiting the island and noting that the pit (from which the earth was extracted) was 'frothing' [9,10].

In short, it is not possible to confirm, if and how, a fungal component was intentionally introduced into the clay, at any particular period. Nevertheless, our experiments are based on this particular hypothesis, namely that a fungal component was indeed intentionally introduced into the clay *and* allowed to co-culture while immersed in water; the purpose of this paper is to proceed to examine the implications of such addition.
