# Supplementary material for: Beneficial modulation of the gut microbiome by leachates of Penicillium purpurogenum in the presence of clays: A model for the preparation and efficacy of historical Lemnian Earth
Source: PLoS One. 2024 Dec 17;19(12):e0313090. doi: 10.1371/journal.pone.0313090 (PMC11651545; doi:10.1371/journal.pone.0313090)

Supporting File S4 - Analysis of Mouse Microbiome Functional Pathways

Figure S4.1: CODA LASSO regression for finding differential MetaCyc pathways between control (PBS solution) samples at day 0 and day 14 of study. (A) β −coefficients returned from CODA-LASSO procedure as two disjoint sets (those that are associated with Pp control d14 are in green , and those that are associated with Pp control d0 are shown in red) (B) Expression levels of these differential MetaCyc pathways where TSS+CLR is Total Sum Scaling followed by Centralised Log Ratio (C) The density plot returned from the CODA-LASSO segregating the two groups and providing a graphical assessment of the classification accuracy (top: actual; bottom: predicted from the procedure using subset of taxa).

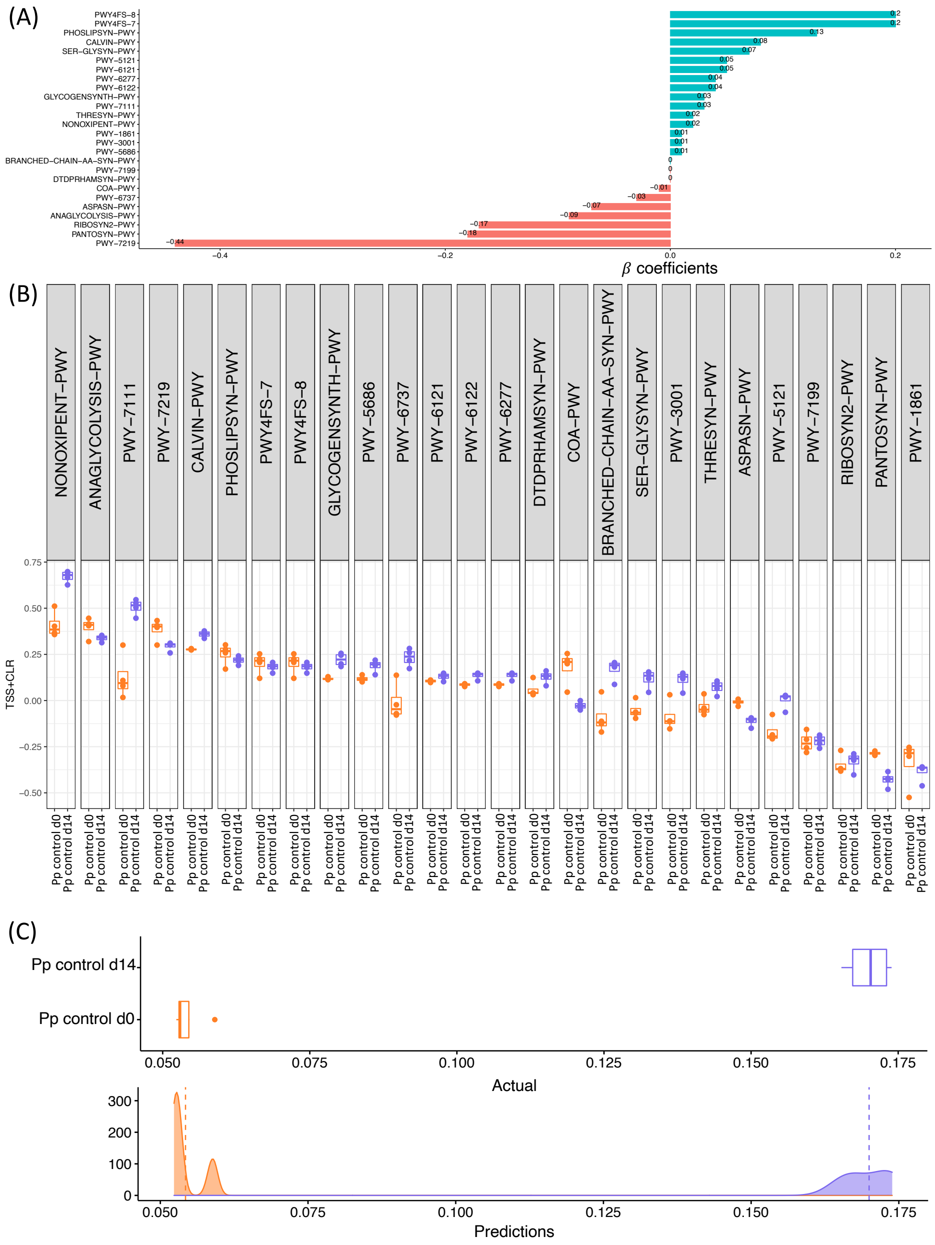

Figure S4.2: CODA LASSO regression for finding differential MetaCyc pathways between Pp+ smectite samples at day 0 and day 14 of study. (A) β −coefficients returned from CODA-LASSO procedure as two disjoint sets (those that are associated with Pp control d14 are in green , and those that are associated with Pp control d0 are shown in red) (B) Expression levels of these differential MetaCyc pathways where TSS+CLR is Total Sum Scaling followed by Centralised Log Ratio (C) The density plot returned from the CODA-LASSO segregating the two groups and providing a graphical assessment of the classification accuracy (top: actual; bottom: predicted from the procedure using subset of taxa).

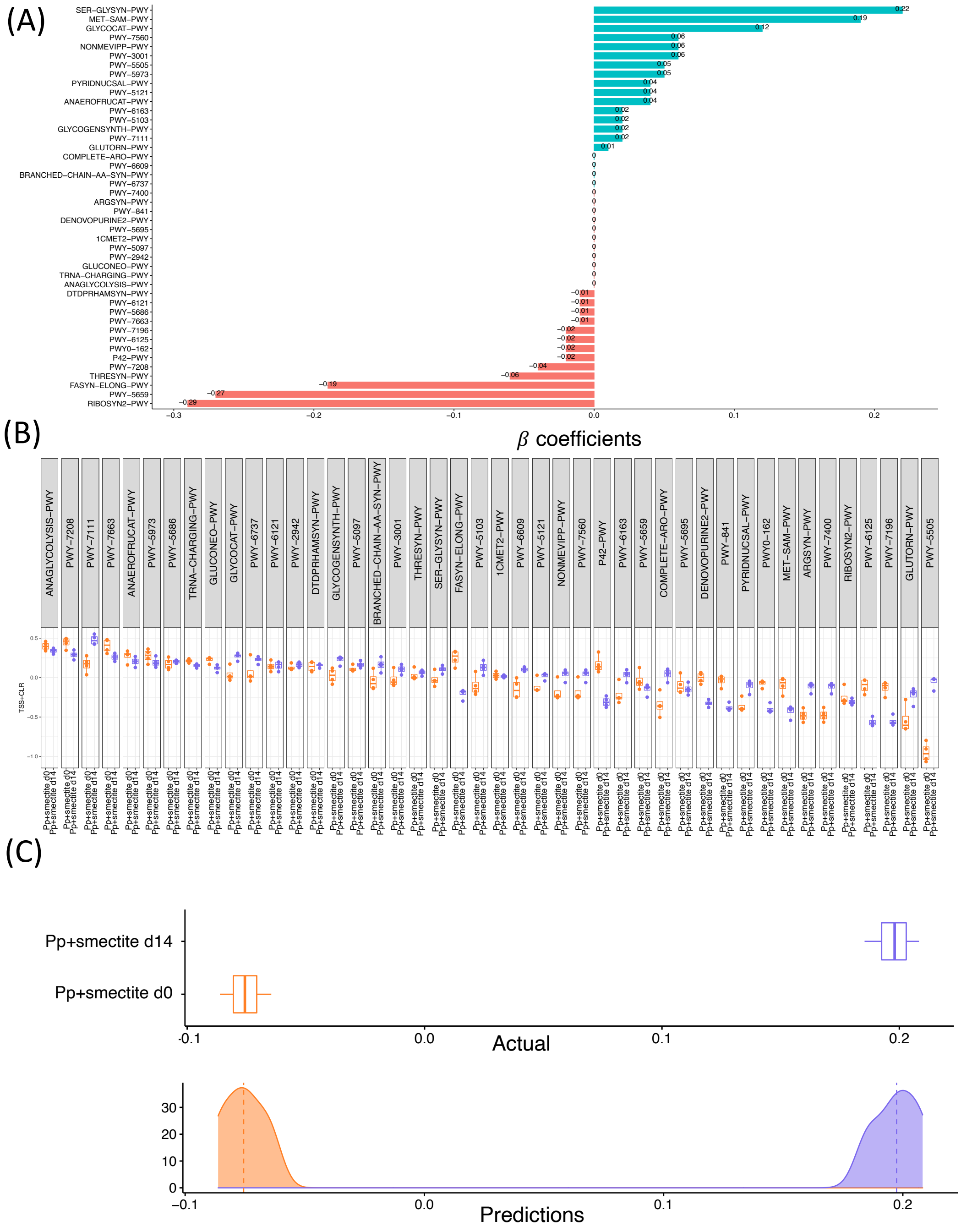

Supplement: S3 File — (PDF) [file pone.0313090.s008.pdf]
